# Diagnosing Lung Cancer: The Complexities of Obtaining a Tissue Diagnosis in the Era of Minimally Invasive and Personalised Medicine

**DOI:** 10.3390/jcm7070163

**Published:** 2018-06-29

**Authors:** Anna E. B. McLean, David J. Barnes, Lauren K. Troy

**Affiliations:** 1 Sydney Medical School, Faculty of Medicine and Health, University of Sydney, Sydney, NSW 2050, Australia; anna.mclean@health.nsw.gov.au (A.E.B.M.); david.barnes@sydney.edu.au (D.J.B.); 2 Department of Respiratory Medicine, Royal Prince Alfred Hospital, Sydney, NSW 2050, Australia

**Keywords:** lung cancer, diagnosis, staging, bronchoscopy, EBUS-TBNA, interventional pulmonology, molecular testing, immunological testing

## Abstract

The role of the respiratory physician in diagnosing lung cancer has increased in complexity over the last 20 years. Adenocarcinoma is now the prevailing histopathological sub-type of non-small cell lung cancer (NSCLC) resulting in more peripheral cancers. Conventional bronchoscopy is often not sufficient to obtain adequate tissue samples for diagnosis. Radiologically guided transthoracic biopsy is a sensitive alternative, but carries significant risks. These limitations have driven the development of complimentary bronchoscopic navigation techniques for peripheral tumour localisation and sampling. Furthermore, linear endobronchial ultrasound with transbronchial needle aspiration (EBUS-TBNA) is increasingly being chosen as the initial diagnostic procedure for those with central lesions and/or radiological evidence of node-positive disease. This technique can diagnose and stage patients in a single, minimally invasive procedure with a diagnostic yield equivalent to that of surgical mediastinoscopy. The success of molecular targeted therapies and immune checkpoint inhibitors in NSCLC has led to the increasing challenge of obtaining adequate specimens for accurate tumour subtyping through minimally invasive procedures. This review discusses the changing epidemiology and treatment landscape of lung cancer and explores the utility of current diagnostic options in obtaining a tissue diagnosis in this new era of precision medicine.

## 1. Introduction

Lung cancer remains the leading cause of cancer-related death in both men and women [1]. Respiratory physicians have a pivotal role in the initial evaluation of patients with suspected lung cancer, with the key goals of obtaining an early tissue diagnosis, accurate staging and assessing the patient’s cardiorespiratory fitness. Changes in the precision and efficacy of lung cancer treatment, the epidemiology of lung cancer, and the tools available for obtaining a tissue diagnosis have significantly increased the complexity of this task over the last 20 years.

## 2. Changing Landscape of Lung Cancer Treatment

Lung cancer can be divided into two broad subgroups: small cell lung cancer (15%) and non-small cell lung cancer (NSCLC, 85%) [2]. Historically, this distinction was considered to be the most important, as a diagnosis of small cell lung cancer precluded surgical management. While NSCLC could be further classified on a resected specimen into adenocarcinoma, squamous cell carcinoma and large cell carcinoma, pursuing this sub-classification on small biopsy specimens prior to treatment was unnecessary as the histological subtype of NSCLC did not influence surgical or chemotherapeutic decisions [3].

The importance of differentiating histological subtypes began with the development of bevacizumab, a humanised anti-vascular endothelial growth factor (VEGF) antibody that improved progression free and overall survival in adenocarcinoma, but could result in severe pulmonary haemorrhage in squamous cell carcinomas [4]. The real push for early differentiation of histological subtypes came with the discovery of epidermal growth factor receptor (EGFR) mutations and subsequent identification of Anaplastic Lymphoma Kinase (ALK) and ROS-1 mutations in tumour specimens. These mutations are almost solely associated with adenocarcinomas and can be treated with targeted therapy to significantly extend progression free survival in advanced disease [3]. More recently, immune checkpoint inhibitors have been shown to be efficacious in all NSCLC [5], introducing new biomarkers to lung cancer diagnosis. This has furthered the need for early adequate tissue, regardless of histological subtype.

## 3. Changing Epidemiology of Lung Cancer

Over the last 50 years, the prevalence of adenocarcinoma has been increasing relative to other lung cancer subtypes, impacting the need to obtain adequate tissue for histological subtyping and molecular testing [6]. The accompanying shift towards more peripherally located primary tumours has increased the difficulty in obtaining diagnostic material [7].

A number of hypotheses have been generated to explain the increasing incidence of adenocarcinoma. Firstly, since the 1950s people have increasingly smoked filtered cigarettes with reduced levels of tar and nicotine. It is theorised that this reduction in nicotine content has resulted in smokers increasing their puff volume, depth and frequency in order to maximise nicotine consumption. This, in combination with filters, which reduce smoke particle size, has resulted in the preferential deposition of carcinogenic smoke within the distal airways favouring the development of peripheral adenocarcinomas [8]. Compounding this phenomenon is the changing composition of cigarette smoke. While the levels of cyclic aromatic hydrocarbons have been reducing, the tobacco specific nitrosamine (TSNA) content of smoke has increased. Nitrosamines have been shown to preferentially induce adenocarcinomas in animal studies [9]. An alternative line of thought is that the increasing incidence of adenocarcinoma may be related to its temporal association with smoking. The relative risk of adenocarcinoma has been found to decline more slowly following smoking cessation than that of squamous cell carcinomas. Feasibly, whilst the significant reduction in smoking rates over the last 50 years has reduced the incidence of squamous cell carcinomas, we are not yet seeing this effect on the incidence of adenocarcinomas [10].

## 4. Obtaining a Tissue Diagnosis

The shift in lung cancer epidemiology from central small cell and squamous cell carcinomas to peripheral adenocarcinomas has impacted the role of traditional bronchoscopy as a first-line diagnostic tool for lung cancer. While tissue sampling with bronchoscopy (through washings, brushings and forceps biopsy) has a diagnostic yield of 88% in large central lesions, the sensitivity declines significantly as lesions become smaller and more distal [11]. In fact, the diagnostic yield of bronchoscopy for lesions <2 cm and in the outer 1/3 of the lung was shown in one study to be as low as 14% [12].

Radiologically guided percutaneous (or transthoracic) needle biopsy is increasingly being employed for the diagnosis of lung cancer. A meta-analysis of published studies suggests the superiority of transthoracic needle biopsy to bronchoscopy in peripheral lesions with a pooled sensitivity of 90% [13]. However, like bronchoscopy, the sensitivity of transthoracic needle biopsy is also affected by lesion size, reducing to 70% in lesions under 1.5 cm [14]. The use of core biopsy over fine needle aspiration (FNA) has not been shown to improve diagnostic sensitivity of malignancy. It has, however, been demonstrated to increase diagnostic specificity in non-malignant lesions [15]. Despite its high sensitivity, caution should be taken in using transthoracic needle biopsy as a “rule out” procedure, as the negative predictive value remains only 70% [16].

There are associated complications with transthoracic needle biopsy. Reported rates of pneumothorax are as high as 60% (with a need for chest drain insertion in up to 15%) [17]. This compares to a pneumothorax rate of less than 2% associated with bronchoscopy [18]. The risk of complication following percutaneous biopsy is increased with smaller, deeper lesions as well as in older, current smokers and those with emphysema [19,20,21]. As a result, the decision to pursue percutaneous biopsies in a frequently high-risk cohort is often a difficult one.

These limitations and the changing topography of lung cancer have driven the recent development of complimentary navigation techniques to improve the ability to localise and therefore sample peripheral lung cancers bronchoscopically.

### 4.1. Radial Probe Endobronchial Ultrasound (EBUS)

This technique utilises a probe that contains an ultrasound able to provide a 360-degree image of surrounding structures. The probe is inserted into the working channel of the bronchoscope and advanced to different segments of the target lobe until the location of the nodule for sampling is determined. Traditional bronchoscopy techniques are then used to perform a biopsy of the located lesion [13]. Impressively, in a large retrospective study, radial probe EBUS was able to localise the target lesion in 96% of cases [22]. A meta-analysis looking at mostly retrospective and small randomised controlled trials of radial probe EBUS reported a pooled diagnostic sensitivity of 73% with a pneumothorax rate of only 1% [23]. Finally, a randomised prospective study performed in an experienced Australian centre demonstrated no statistical difference in diagnostic accuracy between radial probe EBUS (87.5%) and CT guided biopsy (93.3%) but a significantly reduced rate of complications (3% vs. 27%) [24].

### 4.2. Electromagnetic Navigation (EMN)

This involves an electromagnetic tracking system, which uses a position sensor in the tip of a flexible probe deployed through the working channel of the bronchoscope. The position of the sensor is mapped in 3-dimensions, on a real time reconstruction of a previously obtained CT. The position of the sensor can be monitored as it is manoeuvred into the lung periphery and directed based on its position relative to the lesion depicted on the CT image [25]. Several small prospective studies have investigated the benefit of EMN in improving the diagnostic yield of bronchoscopy for small peripheral lesions. In these studies, the average diagnostic yield was 68% with a pneumothorax rate of 4% [13].

More promising was the result of a randomised controlled trial which compared the use of either radial probe EBUS, EMN or the combination of the two, for the diagnosis of peripheral lung lesions. The study found that the diagnostic yield achieved by combining the two techniques increased to 88%, far greater than that for radial probe EBUS (69%) or EMN (59%) alone [26]. The addition of complimentary navigation techniques to traditional bronchoscopy perhaps represents the way forward for the safe and accurate diagnosis of peripheral lung lesions. However, currently, the use of this approach is limited by the restriction of these diagnostic tools to only a few specialist centres.

### 4.3. Cryobiopsy

In addition to navigation techniques which improve the localisation of peripheral lesions bronchoscopically, the use of endobronchial cryobiopsy to enable larger, better quality histopathological specimens may improve the diagnostic yield of bronchoscopy further still. With this technique, a cryoprobe is passed through the instrument channel of the bronchoscope and directed onto the target tissue. Once in position, pressurised gas is passed rapidly through the end of the cryoprobe, to produce an extreme drop in temperature, to as low as −89 degrees Celsius (via the Joule-Thomson effect). The tissue adjacent to the tip of the probe is frozen and can then be removed [27]. In central, endobronchial lesions, cryobiopsy has been shown to be superior to forceps biopsy achieving larger and better preserved biopsy specimens, with increased diagnostic yield (95%) and no increase in adverse events [28,29]. There is however, limited evidence currently on the efficacy of cryobiopsy for peripheral lesions. One small prospective study has demonstrated improved sample size and a small but non-significant increase in diagnostic yield with the addition of cryobiopsy to radial probe EBUS [30]. Table 1 summarises the sensitivities of various diagnostic techniques in lung cancer.

## 5. Concomitant Diagnosis and Staging for Non-Small Lung Cancer

There has been increasing emphasis on mediastinal staging to guide treatment decisions in NSCLC, with clear evidence that primary surgical resection is not beneficial for lung cancers with supraclavicular or contralateral lymph node involvement, or metastasis (Stage IIIb or IV disease) [31]. Linear (or convex probe) endobronchial ultrasound with transbronchial needle aspiration (EBUS-TBNA) is increasingly being chosen as the initial diagnostic procedure for those with central lesions and/or radiological evidence of node-positive disease. Linear EBUS is performed with a flexible bronchoscope which contains a convex transducer at its tip. A 50-degree ultrasound image of the structures underlying the transducer is produced by positioning the probe in direct contact with the wall of a central airway. Once the lymph node or mass is located a TBNA needle is passed through the working channel of the bronchoscope and under real-time ultrasound guidance introduced into the target to obtain a cytological specimen [32].

This technique can diagnose and stage patients in a single, minimally invasive procedure, and is now recommended as first line for staging the mediastinum in all patients with enlarged and/or glucose-avid mediastinal lymph nodes on CT or PET imaging [33]. EBUS-TBNA is also recommended in patients without mediastinal node enlargement or PET avidity, who have a central tumour or enlarged hilar lymph nodes [33], due to false negative rates on PET-CT of up to 25% of these cases [34,35].

Small studies have shown EBUS-TBNA to have at least equivocal sensitivity to standard bronchoscopy in the diagnosis of large central lesions with no increase in adverse events [36,37]. However, the main advantage of EBUS-TBNA is its ability to simultaneously obtain diagnostic tissue and stage the mediastinum. Large prospective trials have now demonstrated the equivalence of EBUS-TBNA to the previous gold standard of surgical mediastinoscopy in staging the mediastinum in terms of sensitivity and yielding the true pathologic stage [38,39]. The safety and tolerability of EBUS-TBNA is also excellent, with a complication rate of less than 1%. A recent study demonstrated that even in the elderly or patients with significant comorbid disease, only 2% poorly tolerated the procedure [40,41]. Methods for optimising the diagnostic yield of EBUS-TBNA have been extensively investigated [42]. The availability of rapid onsite cytologic evaluation and performing at least 3 passes per node have both been demonstrated to improve sensitivity [43,44]. On the other hand, increased needle size, has been shown to have no effect on diagnostic yield [45].

**Figure 1:**
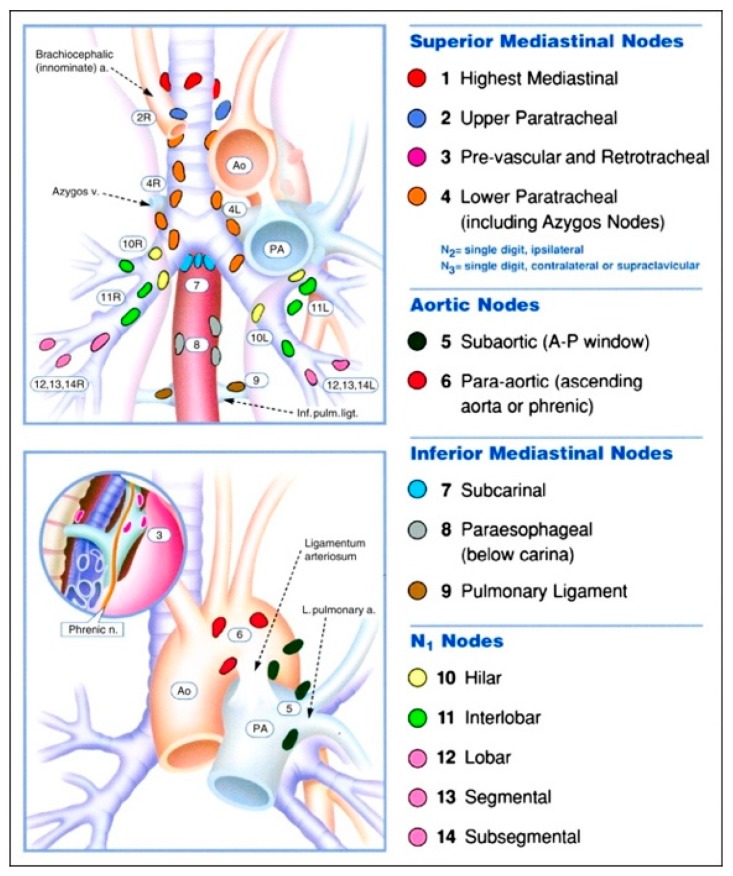
Thoracic lymph node stations. (Permission of [46] requested).

Whilst EBUS-TBNA can sample the majority of lymph node stations it cannot be used for nodes situated beyond the reach of the bronchoscope (stations 3, 5, 6, 8, 9). Most of these however, can be reached via an endoscopic ultrasound (EUS) approach and this has led to the development of a combined EUS and EBUS technique to “completely” stage the mediastinum. Figure 1 demonstrates the locations of the thoracic lymph node stations [46]. To date studies have demonstrated a small improvement in diagnostic sensitivity with the combined approach (91–96%) compared to EUS (89%) or EBUS (84–92%) alone [47,48].

Finally, and perhaps most importantly, the ability of EBUS-TBNA to simultaneously diagnose and stage lung cancer has been shown to significantly reduce time to treatment. In a randomised controlled trial of patients undergoing initial diagnosis comparing EBUS-TBNA to conventional diagnostic staging, the median time to treatment decision was reduced by over 50% or 2 weeks in the EBUS group [49]. Reducing the time to treatment decision can reduce patient anxiety and decrease the risk of intervening deterioration in health, which potentially may limit treatment options.

The high sensitivity and tolerability of EBUS-TBNA for mediastinal staging has left very little role for mediastinoscopy in the diagnosis of lung cancer today. Guidelines now suggest that mediastinoscopy should be reserved for situations where EBUS/EUS is not accessible or when very high clinical suspicion of mediastinal involvement remains despite negative EBUS/EUS [33]. 

## 6. Specialised Testing on Small Specimens

### 6.1. Molecular Testing

Molecular driver mutations are an important therapeutic target in advanced NSCLC. In a recent large meta-analysis, EGFR mutations were present in 32.3% of 115,815 NSCLC tumours. The prevalence was significantly higher in the Asian population (38.4%) compared to patients from America (24.4%) or Europe (14.1%). EGFR mutations were also more commonly detected in females, non-smokers and adenocarcinomas [50]. Comparatively, ALK and ROS1 gene translocations are far less common, identified in 1–2% and 4–5% of NSCLC, respectively. ALK and ROS1 rearrangements are found almost exclusively in adenocarcinomas and patients tend to be younger, never-smokers. Importantly ALK translocations have a male preponderance [51,52]. As a result, molecular testing is now indicated in all patients with advanced stage adenocarcinoma to assess for these three targetable mutations [53,54].

Minimally invasive diagnostic techniques are therefore chiefly beneficial to the patient if adequate tissue can be provided to enable detailed molecular characterisation. The need for repeat procedures to obtain sufficient tissue would potentially negate the minimally invasive aspect of the procedure. This is, however, sometimes necessary, particularly when considering second- and third-line therapeutic options.

Multiple studies have now investigated the viability of different diagnostic techniques in obtaining adequate material for molecular testing. Although direct comparisons are limited, cytological specimens and small histopathological specimens appear to perform similarly, with high feasibility of molecular testing demonstrated, irrespective of the diagnostic modality [55,56,57]. The one exception was exfoliative cytology samples, (brushings and washings) which did demonstrate reduced adequacy for molecular testing in two small trials [58,59]. Perhaps unexpectedly, FNA has been shown to be comparable to core biopsy for molecular testing following radiologic transthoracic needle biopsy [60]. Finally, one study has directly compared the feasibility of molecular testing in core needle biopsy versus forceps endobronchial biopsy versus EBUS-TBNA. Whilst the adequacy of material for EGFR testing was 100% across all three modalities, EBUS-TBNA was found to generate the highest volume of RNA for molecular testing, perhaps signalling superiority [61].

### 6.2. Immunologic Testing

Immunotherapy using antibodies to inhibit the binding of Programmed Death Ligand 1 (PD-L1) to Programmed Death Receptor 1 (PD-1) has significantly changed the treatment landscape for advanced NSCLC. The PD-1 pathway is a key negative regulator of the anti-tumour immune response. Binding of PD-L1 expressed on tumour cells to PD-1, suppresses the T cell response by inhibiting proliferation and cytokine production. Inhibiting this pathway releases the host immune response, promoting enhanced anti-tumour activity [62]. Cancers with high levels of somatic gene mutations, such as NSCLC, are particularly vulnerable to this approach due to their elevated expression of tumour specific antigens and thus increased immunogenicity [63]. Two PD-1 inhibitors, nivolumab and pembrolizumab have been shown to improve overall survival in previously treated advanced NSCLC compared to chemotherapy and are now approved for use in this setting [64,65,66]. More recently pembrolizumab was approved for use in the first line setting for advanced NSCLC after demonstrating superiority to chemotherapy [5].

While early studies of PD-1 inhibition demonstrated a significant and durable effect, a relatively small response rate of approximately 20% was noted [67]. As a result, a biomarker was needed to select out those patients who were more likely to benefit from PD-1 inhibition. Multiple studies including a large meta-analysis have now demonstrated that PD-L1 expression predicts responsiveness to PD-1 inhibitors in several tumour types. Importantly, PD-L1 expression has not been demonstrated to influence treatment response in squamous cell carcinomas [68]. Regardless, PD-L1 expression evaluated by immunohistochemistry (IHC) is now widely used to direct the use of PD-1 inhibitors in patients with advanced NSCLC. However, the suitability of PD-L1 expression as a biomarker to inform treatment options in NSCLC is controversial, as its testing remains complicated by multiple issues [69].

Currently there is no clear definition of positive PD-L1 staining by IHC. Across positive studies, cut offs have ranged from >1% to >50% tumour cells stained. Determining this threshold could dramatically affect the population able to access these new therapies, as while only 16–40% of NSCLCs have PD-L1 expression > 50%, over 60% have expression > 1% [62]. Furthermore, at this stage a standardized assay for PD-L1 scoring does not exist. Several different assays, usually associated with a particular therapeutic agent, can be used. Studies comparing these assays have demonstrated significant discordance in positive staining [70]. Finally, unlike molecular markers, PD-L1 expression is not an intrinsic property of the tumour, but rather part of a dynamic response to the tumour microenvironment. As such, significant heterogeneity of PD-L1 expression has been demonstrated both within tumours and across different sites of disease. In one study PD-L1 expression in primary tumours differed from nodal metastases in 38% of cases [70].

It is possible that tumour heterogeneity could result in an increased risk of false negatives in small biopsy and cytology samples. Currently, there are no assays recommended for use on cytology specimens due to the lack of technical validation, use in clinical trials and development of a specific scoring algorithm [69]. However, the increased use of minimally invasive diagnostic techniques has driven increasing evidence that PD-L1 IHC testing on cytology specimens is both feasible and comparable to larger biopsies. A recent prospective study comparing cytological versus small biopsy versus surgical specimens in over 180 patients demonstrated no difference in the adequacy of tissue for PD-L1 staining in cytological samples compared to both small biopsy and surgical specimens [71].

## 7. Further Research

This review addresses the considerations surrounding obtaining a tissue diagnosis and sufficient material for molecular testing and immunotherapy, focusing on NSCLC where the most rapid developments have occurred. However, there are many other areas within lung cancer diagnosis that are advancing at a similar pace and require their own review to adequately describe. This includes the role of imaging, in particular the impact of PET-CT in staging lung cancer as well as advancements in CT technology such as radiomics and low dose scanning in order to improve the accuracy and safety of screening and diagnosis. The emergence of ‘Liquid biopsies’ which takes the minimally invasive diagnosis of cancer to the next level is another area of rapid development fuelled by the rise of targeted therapies. Liquid biopsies enable the diagnosis of lung cancer through the detection of circulating biomarkers including exosomes, circulating tumour cells and circulating DNA in blood [72]. This represents an exciting area as it could feasibly enable not only the diagnosis of lung cancer but also comprehensive genomic profiling and monitoring of treatment response with a simple blood test.

## 8. Conclusions

The task of diagnosing lung cancer has dramatically increased in difficulty and complexity over the last 20 years, driven by changing epidemiology and the rapid expansion and personalisation of treatment options in NSCLC. Whilst a small sample to distinguish between small cell and NSCLC was previously sufficient, the initial diagnostic procedure for lung cancer should now ideally simultaneously provide tissue for histopathological diagnosis, molecular testing and staging. Diagnostic techniques have adapted and developed in response to these changing conditions and there is now an arsenal of minimally invasive tools for lung cancer diagnosis. The utility and safety of these procedures vary greatly depending on the characteristics of both the patient and the tumour, and as a result the respiratory physician is faced with an increasingly difficult and individualised decision when planning the best diagnostic approach. A multidisciplinary approach to the diagnosis of lung cancer, including input from the respiratory proceduralist, has become increasingly important in ensuring the most efficient, low-risk and accurate diagnostic process for patients.

## Figures and Tables

**Table 1:** Summary of the sensitivities of the various diagnostic modalities for the diagnosis and staging of lung cancer [13,29,30].

Diagnostic Stage	Diagnostic Modality	Sensitivity (%)	
		Central	Peripheral
Initial Diagnosis	Sputum cytology	71	49
	Bronchoscopy	88	78
	Washings	47	43
	Brushings	56	54
	Biopsy	74	57
	Radiologically guided transthoracic biopsy	-	90
	Radial probe EBUS	-	73
	Electromagnetic navigation	-	68
	Cryobiopsy	95	74
	Linear EBUS TBNA	82	-
Mediastinal Staging	Bronchoscopic TBNA	78	
	Linear EBUS TBNA	89	
	EUS	89	
	EUS + EBUS	91	
	Video assisted surgical mediastinoscopy	89	

EBUS = Endobronchial Ultrasound; TBNA = Transbronchial Needle Aspiration; EUS = Endoscopic Ultrasound.

## References

[1] Ferlay J., Soerjomataram I., Ervik M., Dikshit R., Eser S., Mathers C., Rebelo M., Parkin D.M., Forman D., Bray F. (2013). GLOBOCAN 2012v1.0, Cancer Incidence and Mortality Worldwide: IARC Cancer Base No. 11.

[2] Reck M., Rabe K. (2017). Precision diagnosis and treatment for advanced non-small-cell lung cancer. N. Engl. J. Med..

[3] Moreira A.L., Travis W.D., Moreira A.L., Saqi A. (2015). Histological Classification and Its Need for Treatment of Lung Cancer. Diagnosing Non-Small Cell Carcinoma in Small Biopsy and Cytology.

[4] Johnson D.H., Fehrenbacher L., Novotny W.F., Herbst R.S., Nemunaitis J.J., Jablons D.M., Langer C.J., DeVore R.F., Gaudreault J., Damico L.A. (2004). Randomized phase II trial comparing bevacizumab plus carboplatin and paclitaxel with carboplatin and paclitaxel alone in previously untreated locally advanced or metastatic non-small- cell lung cancer. J. Clin. Oncol..

[5] Reck M., Rodriguez-Abreu D., Rao S., Hui R., Csőszi T., Fülöp A., Gottfried M., Peled N., Tafreshi A., Cuffe S. (2016). Pembrolizumab versus chemotherapy for PD-L1-positive non-small-cell lung cancer. N. Engl. J. Med..

[6] Khuder S. (2001). Effect of cigarette smoking on major histological types of lung cancer: A meta-analysis. Lung Cancer.

[7] Brooks D.R., Austin J.H.M., Heelan R.T., Ginsberg M.S., Shin V., Olson S.H., Muscat J.E., Stellman S.D. (2005). Influence of type of cigarette on peripheral versus central lung cancer. Cancer Epidemiol. Prev. Biomark..

[8] Wynder E.L., Muscate J.E. (1995). The changing epidemiology of Smoking and Lung Cancer Histology. Environ. Health Perspect..

[9] Alberg A.J., Brock M.V., Samet J.M. (2005). Epidemiology of lung cancer: Looking to the future. J. Clin. Oncol..

[10] Charloux A., Quoix E., Kreisman H., Small D., Pauli G., Kreisman H. (1997). The increasing incidence of lung adenocarcinoma: Reality or artefact? A review of the epidemiology of lung adenocarcinoma. Int. J. Epidemiol..

[11] Shreiber G., McCrory D.C. (2003). Performance characteristics of different modalities for diagnosis of suspected lung cancer: Summary of published evidence. Chest.

[12] Baalini W.A., Reinoso M.A., Gorin A.B., Sharafkaneh A., Manian P. (2000). Diagnostic yield of fiberoptic bronchoscopy in evaluating solitary pulmonary nodules. Chest.

[13] Rivera P., Mehta A.C., Wahidi M.M. (2013). Establishing the diagnosis of lung cancer: Diagnosis and management of lung cancer: American College of Chest Physicians evidence-based clinical practice guidelines. Chest.

[14] Li H., Boiselle P.M., Shepard J.O., Trotman-Dickenson B., McLoud T.C. (1996). Diagnostic accuracy and safety of CT-guided percutaneous needle aspiration biopsy of the lung: Comparison of small and large pulmonary nodules. Am. J. Roentgenol..

[15] Klein J.S., Salomon G., Stewart E.A. (1996). Transthoracic needle biopsy with a coaxially placed 20-gauge automated cutting needle: Results in 122 patients. Radiology.

[16] Zarbo R.J., Fenoglio-Preiser C.M. (1992). Interinstitutional database for comparison of performance in lung fine-needle aspiration cytology. A College of American Pathologists Q-Probe Study of 5264 cases with histologic correlation. Arch. Pathol. Lab. Med..

[17] Manhire A., Charig M., Clelland C., Gleeson F., Miller R., Moss H., Pointon K., Richardson C., Sawicka E. (2003). Guidelines for radiological guided lung biopsy. Thorax.

[18] Ost D.E., Ernst A., Lei X., Kovitz K.L., Benzaquen S., Diaz-Mendoza J., Greenhill S., Toth J., Feller-Kopman D., Puchalski J. (2016). Diagnostic yield and complications of bronchoscopy for peripheral lung lesions: Results of the AQuIRE registry. Am. J. Respir. Crit. Care Med..

[19] Kazerooni E.A., Lim F.T., Mikhail A., Martinez F.J. (1996). Risk of pneumothorax in CT-guided transthoracic needle aspiration biopsy of the lung. Radiology.

[20] Yeow K.M., See L.C., Lui K.W., Lin M.C., Tsao T.C., Ng K.F., Liu H.P. (2001). Risk factors for pneumothorax and bleeding after CT-guided percutaneous coaxial cutting needle biopsy of lung lesions. J. Vasc. Interv. Radiol..

[21] Wiener R.S., Schwartz L.M., Woloshin S., Welch H.G. (2011). Population-based risk for complications after transthoracic needle lung biopsy of a pulmonary nodule: An analysis of discharge records. Ann. Inter. Med..

[22] Chen A., Chenna P., Loiselle A., Massoni J., Mayse M., Misselhorn D. (2014). Radial probe endobronchial ultrasound for peripheral pulmonary lesions: A 5-year Institutional experience. Ann. Am. Thorac. Soc..

[23] Steinfort D.P., Khor Y.H., Manser R.L., Irving L.B. (2011). Radial probe endobronchial ultrasound for the diagnosis of peripheral lung cancer: Systematic review and meta-analysis. Eur. Respir. J..

[24] Steinfort D.P., Vincent J., Heinze S., Antippa P., Irving L.B. (2011). Comparative effectiveness of radial probe endobronchial ultrasound versus CT-guided needle biopsy for evaluation of peripheral pulmonary lesions: A randomized pragmatic trial. Respir. Med..

[25] Becker H.D., Herth F., Ernst A., Schwarz Y. (2005). Bronchoscopic biopsy of peripheral lung lesions under electromagnetic guidance: A pilot study. J. Bronchol. Interv. Pulmonol..

[26] Eberhardt R., Anantham D., Ernst A., Feller-Kopman D., Herth F. (2007). Multimodality bronchoscopic diagnosis of peripheral lung lesions: A randomized controlled trial. Am. J. Respir. Crit. Care Med..

[27] ERBE Flexible Cryoprobes for Bronchoscopic Diagnosis and Treatment. NICE Guidelines 2015. www.nice.org.UK.

[28] Hetzel J., Hetzel M., Hasel C., Moeller P., Babiak A. (2008). Old meets modern: The use of traditional cryoprobes in the age of molecular biology. Respiration.

[29] Hetzel J., Eberhardt R., Herth F.J.F., Petermann C., Reichle G., Freitag L., Dobbertin I., Franke K.J., Stanzel F., Beyer T. (2012). Cryobiopsy increases the diagnostic yield of endobronchial biopsy: A multicentre trial. Eur. Respir. J..

[30] Schuhmann M., Bostanci K., Bugalho A., Warth A., Schnabel P.A., Herth F.J., Eberhardt R. (2014). Endobronchial Ultrasound-guided cryobiopsies in peripheral pulmonary lesions: A feasibility study. Eur. Respir. J..

[31] Ramnath N., Dilling T.J., Harris L.J., Arennberg D.A. (2013). Treatment of stage III non-small cell lung cancer: Diagnosis and management of lung cancer: American College of Chest Physicians evidence-based clinical practice guidelines. Chest.

[32] Gomez M., Silvestri G.A. (2009). Endobronchial ultrasound for the diagnosis and staging of lung cancer. Proc. Am. Thorac. Soc..

[33] Silvestri G.A., Gonzalez A.V., Jantz M.A., Detterbeck F.C. (2013). Methods for staging non-small cell lung cancer: Diagnosis and management of lung cancer: American College of Chest Physicians evidence-based clinical practice guidelines. Chest.

[34] Fischer B., Lassen U., Mortensen J., Larsen S., Loft A. (2009). Pre-operative staging of lung cancer with combined PET-CT. N. Engl. J. Med..

[35] Cerfolio R.J., Bryant A.S., Ojha B., Eloubeidi M. (2005). Improving the inaccuracies of clinical staging of patients with NSCLC: A prospective Trial. Ann. Thorac. Surg..

[36] Tournoy K.G., Rintoul R.C., Van Meerbeeck J.P., Annema J.T. (2009). EBUS-TBNA for the diagnosis of central parenchymal lung lesions not visible at routine bronchoscopy. Lung Cancer.

[37] Vaidya P.J., Kate A.H., Yasufuku K., Chhajed P.N., Chhajed P.N. (2015). Endobronchial ultrasound-guided transbronchial needle aspiration in lung cancer diagnosis and staging. Expert Rev. Respir. Med..

[38] Yasufuku K., Pierre A., Darling G., de Perrot M., Waddell T., Johnston M., da Cunha Santos G., Geddie W., Boerner S., Le L.W. (2011). A prospective controlled trial of endobronchial ultrasound-guided transbronchial needle aspiration compared with mediastinoscopy for mediastinal lymph node staging of lung cancer. J. Thorac. Cardiovasc..

[39] Um S.W., Kim H.K., Jung S.H., Han J., Lee K.J., Park H.Y., Choi Y.S., Shim Y.M., Ahn M.J., Park K. (2015). Endobronchial ultrasound versus mediastinoscopy for mediastinal nodal staging of non-small-cell lung cancer. J. Thorac. Oncol..

[40] Gu P., Zhao Y.-Z., Jian L.-Y., Zhang W., Xin Y., Han B.-H. (2009). Endobronchial ultrasound-guided transbronchial needle aspiration for staging of lung cancer: A systematic review and meta-analysis. Eur. J. Cancer.

[41] Evison M., Crosbie P.A.J., Martin J., Booton R. (2014). EBUS-TBNA in elderly patients with lung cancer safety and performance outcomes. J. Thorac. Oncol..

[42] Van Der Heijden E.H., Casal R.F., Trisolini R., Steinfort D.P., Hwangbo B., Nakajima T., Guldhammer-Skov B., Rossi G., Ferretti M., Herth F.F. (2014). Guidelines for the acquisition and preparation of conventional and endobronchial ultrasound-guided transbronchial needle aspiration specimens for the diagnosis and molecular testing of patient with known or suspected lung cancer. Respiration.

[43] Bulman W., Saqi A., Powerll C.A. (2012). Acquisition and processing of Endobronchial ultrasound guided transbronchial needle aspiration specimens in the era of targeted lung cancer chemotherapy. Am. J. Respir. Crit. Care Med..

[44] Lee H.S., Lee G.K., Lee H.S., Kim M.S., Lee J.M., Kim H.Y., Nam B.H., Zo J.L., Hwangbo B. (2008). Real-time endobronchial ultrasound-guided transbronchial needle aspiration in mediastinal staging of non-small cell lung cancer: How many aspirations per target lymph node station?. Chest.

[45] Oki M., Saka H., Kitagawa C., Kogure Y., Mu-rata N., Ichihara S., Moritani S., Ando M. (2011). Randomized study of 21-gauge versus 22-gauge endobronchial ultrasound-guided transbronchial needle aspiration needles for sampling histology specimens. J. Bronchol. Interv. Pulmonol..

[46] Mountain C.F., Dresler C.M. (1997). Regional lymph node classification for lung cancer staging. Chest.

[47] Hwangbo B., Lee G.K., Lee H.S., Lim K.Y., Lee S.H., Kim H.Y., Lee H.S., Kim M.S., Lee J.M., Nam B.H. (2010). Transbronchial and transesophageal fine-needle aspiration using an ultrasound bronchoscope in mediastinal staging of potentially operable lung cancer. Chest.

[48] Herth F.J., Krasnik M., Kahm N., Eberhardt R., Ernst A. (2010). Combined endoscopic-endobronchial ultrasound-guided fine-needle aspiration of mediastinal lymph nodes through a single bronchoscope in 150 patients with suspected lung cancer. Chest.

[49] Navani N., Nankivell M., Lawrence D.R., Lock S., Makker H., Baldwin D.R., Stephens R.J., Parmar M.K., Spiro S.G., Morris S. (2015). Lung cancer diagnosis and staging with endobronchial ultrasound-guided transbronchial needle aspiration compared with conventional approaches: An open-label, pragmatic, randomised controlled trial. Lancet Respir. Med..

[50] Zhang Y., Yuan J., Tang J. (2006). The prevalence of EGFR mutation in patients with non-small cell lung cancer: A systematic review and meta-analysis. Oncotarget.

[51] Davies K.D., Doevele R.C. (2013). Molecular Pathways—ROS1 Fusion Proteins in Cancer. Clin. Cancer Res..

[52] Chia P.L., Mitchell P., Dobrovic A., Jonh T. (2014). Prevalence and natural history of ALK positive non-small-cell lung cancer and the clinical impact of targeted therapy with ALK inhibitors. Clin. Epidemiol..

[53] Cancer Council Australia Lung Cancer Guidelines Working Party (2017). Clinical Practice Guidelines for the Treatment of Lung Cancer.

[54] Lindeman N.I., Cagle P.T., Beasley M.B., Chitale D.A., Dacic S., Giaccone G., Jenkins R.B., Kwiatkowski D.J., Saldivar J.S., Squire J. (2013). Molecular testing guideline for selection of lung cancer patients for EGFR and ALK tyrosine kinase inhibitors: Guideline from the College of American Pathologists, International Association for the Study of Lung Cancer, and Association for Molecular Pathology. J. Thorac. Oncol..

[55] Ofiara L.M., Navasakulpong A., Beaudoin S., Gonzalez A.V. (2014). Optimising tissue sampling for the diagnosis, subtyping and molecular analysis of cancer. Front. Oncol..

[56] Wang S., Yu B., Ng C.C., Mercorella B., Selinger C.I., O’Toole S.A., Cooper W.A. (2015). The suitability of small biopsy and cytological specimens for EGFR and other mutation testing in non-small cell lung cancer. Transl. Lung Cancer Res..

[57] Oki M., Yatabe Y., Saka H., Moritani S. (2015). Feasibility and accuracy of molecular testing specimens obtained from small biopsy forceps: Comparison with the results of surgical specimens. Respiration.

[58] Allegrini S., Antona J., Mezzapelle R., Miglio U., Paganotti A., Veggiani C., Frattini M., Monga G., Balbo P., Boldorini R. (2012). Epidermal growth factor receptor gene analysis with a highly sensitive molecular assay in routine cytological specimens of lung adenocarcinoma. Am. J. Clin. Pathol..

[59] Rekhtman N., Brandt S., Sigel C., Moreira A.L. (2011). Suitability of thoracic cytology for new therapeutic paradigms in Non-small cell lung carcinoma. High accuracy of tumour subtyping and feasibility of EGFR and KRAS molecular testing. J. Thorac. Oncol..

[60] Coley S., Crapanzano J.P., Saqi A. (2015). FNA, Core biopsy or both for the diagnosis of lung carcinoma. Obtaining sufficient tissue for a specific diagnosis and molecular testing. Cancer Cytopathol..

[61] Schmid-Bindert G., Wang Y., Jiang H., Zhou C. (2013). EBUS-TBNA Provides highest RNA yield for multiple biomarker testing from routinely obtained small biopsies in non-small cell lung cancer patients—Comparative study of three different minimal invasive sampling methods. PLoS ONE.

[62] Yu H., Boyle T., Zhou C., Rimm D.L., Hirsch F.R. (2016). PD-L1 expression lung cancer. J. Thorac. Oncol..

[63] Hamanishi J., Mandai M., Matsumura N., Abiko K., Baba T., Konishi I. (2016). PD-1/PD-L1 blockade in cancer treatment: Perspectives and issues. Int. J. Clin. Oncol..

[64] Borghaei H., Paz-Ares L., Horn L., Spigel D.R., Steins M., Ready N.E., Chow L.Q., Vokes E.E., Felip E., Holgado E. (2015). Nivolumab versus docetaxel in advanced nonsquamous non-small-cell lung cancer. N. Engl. J. Med..

[65] Brahmer J., Reckamp K.L., Baas P., Crinò L., Eberhardt W.E., Poddubskaya E., Antonia S., Pluzanski A., Vokes E.E., Holgado E. (2015). Nivolumab versus docetaxel in advanced squamous-cell non-small-cell lung cancer. N. Engl. J. Med..

[66] Herbst R.S., Baas P., Kim D.W., Felip E., Pérez-Gracia J.L., Han J.Y., Molina J., Kim J.H., Arvis C.D., Ahn M.J. (2016). Pembrolizumab versus docetaxel for previously treated, PD-L1-positive, advanced non-small-cell lung cancer (KEYNOTE-010): A randomised controlled trial. Lancet.

[67] Herbst R.S., Soria J., Kowanetz M., Hodi F.S. (2014). Predictive correlates of response to the anti-PD-L1 antibody MPDL3280A in cancer patients. Nature.

[68] Gandini S., Massi D., Mandala M. (2001). Pd-L1 expression in cancer patients receiving anti PD-1/PD-L1 antibodies: A systematic review and meta-analysis. Crit. Rev. Oncol. Hematol..

[69] Teixido C., Vilarino N., Reyes R., Reguart N. (2018). PD-L1 expression testing in non-small cell lung cancer. Ther. Adv. Med. Oncol..

[70] Matthen M., Safyan R., Shu C.A. (2017). PD-L1 as a biomarker in NSCLC: Challenges and future directions. Ann. Transl. Med..

[71] Heymann J.J., Bulman W.A., Swinarski D., Pagan C.A., Saqi A. (2017). PD-L1 expression in non-small cell lung carcinoma: Comparison among cytology, small biopsy, and surgical resection specimens. Cancer Cytopathol..

[72] Zhang Y., Zhou Q., Wu Y. (2017). The emerging roles of NGS-based liquid biopsy in non-small cell lung cancer. J. Hematol. Oncol..

